# A Case of Thyrotoxic Periodic Paralysis: "I Can't Move!"

**DOI:** 10.7759/cureus.34301

**Published:** 2023-01-28

**Authors:** Arjun Basnet, Nitasha Goyal, Kripa Tiwari, Sajog Kansakar, Sudarshan Gautam

**Affiliations:** 1 Internal Medicine, Maimonides Medical Center, New York City, USA; 2 Internal Medicine, State University of New York (SUNY) Downstate College of Medicine, New York City, USA

**Keywords:** graves' disease, asian ethnicity, qt interval prolongation, paralysis, thyrotoxic periodic paralysis

## Abstract

Thyrotoxic periodic paralysis (TPP) is a form of hypokalemic periodic paralysis associated with hyperthyroidism. It is characterized by hypokalemia associated with acute proximal symmetrical lower limb weakness and can progress to involve all four limbs and the respiratory musculature. We present a case of a 27-year-old Asian male with recurrent attacks of weakness in all four extremities. A subsequent diagnosis of thyrotoxic periodic paralysis was made, which was secondary to a previously undiagnosed Grave’s disease. TPP should be a differential in a young male of Asian ethnicity who presents to the hospital with acute onset of paralysis.

## Introduction

Thyrotoxic periodic paralysis (TPP) is a form of hypokalemic periodic paralysis associated with hyperthyroidism. It is characterized by hypokalemia associated with acute proximal symmetrical lower limb weakness and can progress to involve all four limbs and the respiratory musculature. TPP is common in East Asian populations, with an incidence of approximately 2% [1]. Despite thyrotoxicosis affecting females nine times more than males, TPP occurs in males in ratios ranging from 17:1 to 70:1 [2, 3]. We present a case of TPP in a young male who presented to the hospital because of an inability to move his upper and lower extremities upon awakening from sleep.

## Case presentation

A 27-year-old Asian male with no known co-morbidities presented to the hospital because of an inability to move his upper and lower extremities since awakening from sleep in the morning and was accompanied by difficulty breathing. The patient had similar episodes in the past when he developed sudden onset weakness with the inability to move his arms and legs with spontaneous resolution. Prior attacks were precipitated by drinking beer and physical exertion. Previous episodes were mild, lasted a few minutes, and resolved spontaneously. He denied bowel or bladder incontinence, trauma, fever, recent illness, headaches, vision changes, dysarthria, loss of consciousness, drug use, fever, or any recent illness.

**Figure 1:**
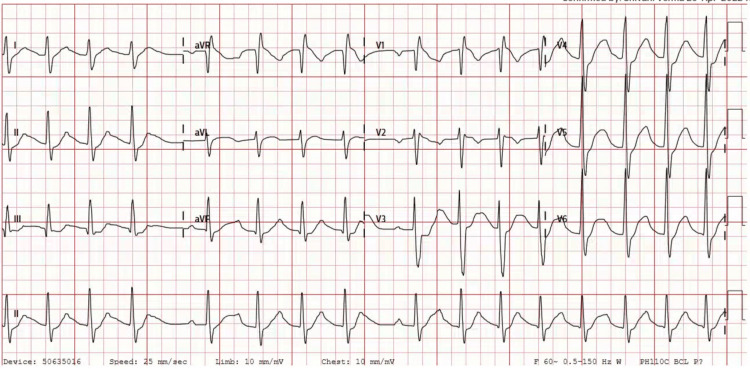
Electrocardiogram showing prolonged QT interval of 503 ms

He was afebrile, and his vitals were within normal limits. Neurological examination was remarkable for power of 0/5 in bilateral upper and lower extremities. There were no sensory deficits. Physical exams of other systems were unremarkable. Electrocardiogram showed a prolonged QTc interval of 503 ms (Figure 1). Lab studies are summarized in Table 1 and were significant for electrolyte abnormalities, including hypokalemia, hypomagnesemia, and hypophosphatemia. Arterial blood gas revealed normal acid-base status. Creatine phosphokinase was within normal limits. Other significant findings included low TSH, high serum-free T4, and elevated serum-free T3.

**Table 1:** Laboratory investigations TSH - thyroid-stimulating hormone; T4 - thyroxine; T3 - triiodothyronine

Laboratory Parameters	Value	Reference Range
Potassium (mmol/L)	1.3	3.4-4.8
Magnesium (mg/dL)	1.5	1.6-2.2
Phosphorus (mg/dL)	1.5	2.2-5.5
Creatine phosphokinase (IU/L)	130	59-367
TSH (mIU/ml)	<0.01	0.5- 5.0
Serum-freeT4 (ng/dL)	4.27	0.58-1.64
Serum-free T3 (ng/dL)	14.09	2.53-3.87
Thyroid-stimulating immunoglobulin (IU/L)	4.14	0.00-0.55
Thyroid peroxidase antibody (mIU/ml)	240	<34.9

**Figure 2:**
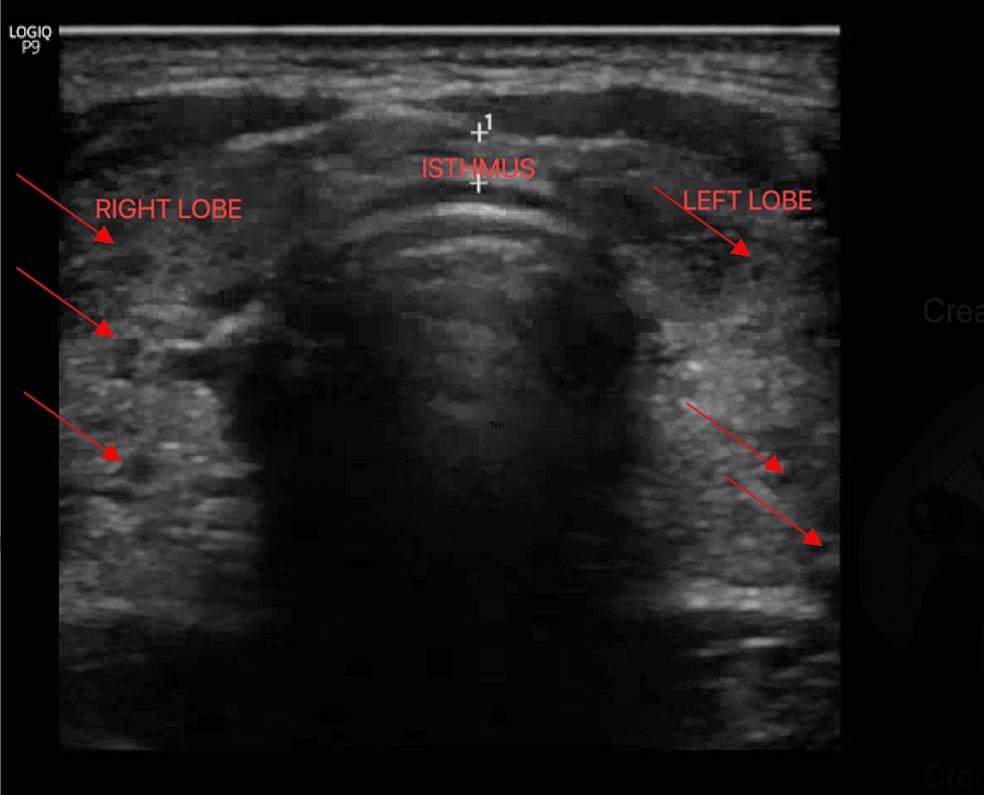
Ultrasound of the thyroid gland showing enlarged right and left lobes with arrows representing bilateral sub-centimeter thyroid nodules

**Figure 3:**
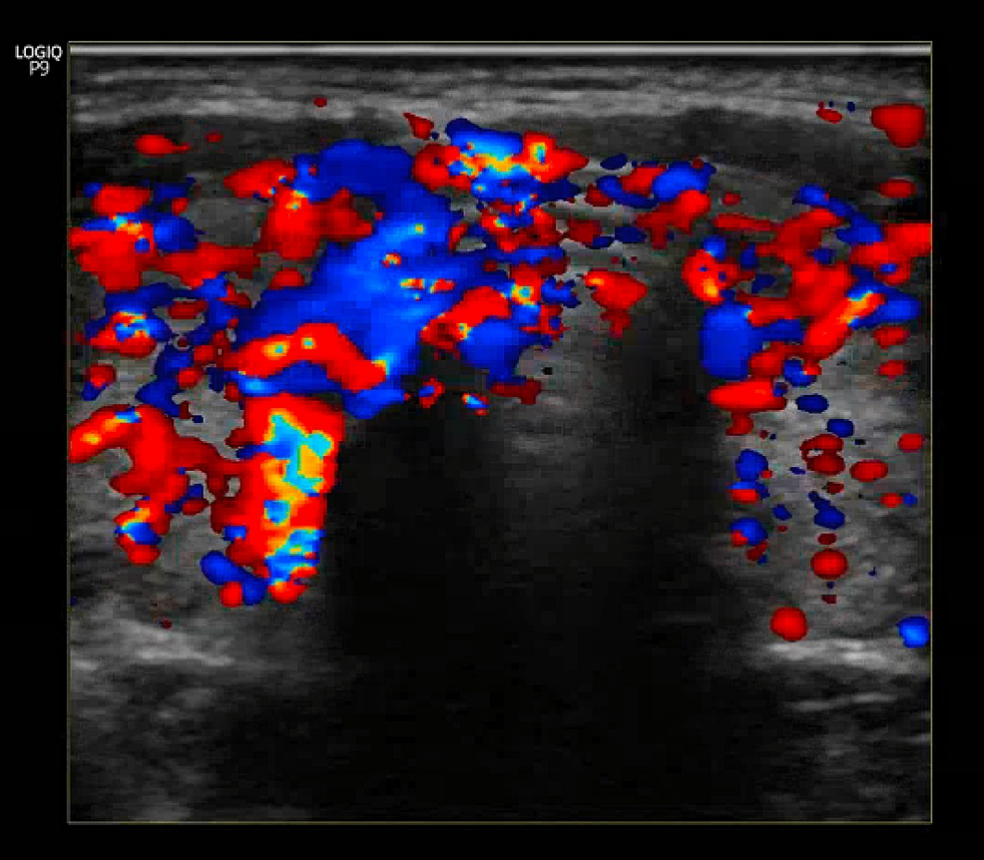
Ultrasound of the thyroid gland showing diffuse increased vascular flow throughout the gland

A provisional diagnosis of TTP was made, and the patient was admitted to the medical intensive care unit (ICU) for aggressive repletion of electrolytes. Electrolytes (K and Mg) were supplemented, and Intravenous beta-blocker(propranolol) and methimazole were also administered. His symptoms improved rapidly within hours of therapy following electrolyte replacement and propranolol. Upon further workup, thyroid-stimulating immunoglobulin was elevated, and thyroid peroxidase antibody was also elevated (Table 1), confirming a new diagnosis of Grave's disease. Ultrasound of the thyroid revealed enlarged right and left lobes of the thyroid gland, bilateral sub-centimeter thyroid nodules, and diffuse increased vascular flow throughout the thyroid gland (Figure 2,3). He was discharged home on methimazole and propranolol with endocrinology follow-up for further workup and management of hyperthyroidism.

## Discussion

Thyrotoxic periodic paralysis is characterized by a thyroid hormone-induced intracellular shift of potassium, leading to muscle weakness and paralysis. It typically affects Asian males between the ages of 20-40 [4, 5, 6]. One study showed that 65% of TPP patients had initial thyrotoxic symptoms and 60% were clinically thyrotoxic at presentation [5]. Our patient at presentation had abnormal TSH, FT4, and FT3; however, he did not have any thyrotoxic symptoms, and his vitals were stable.

Precipitation of attacks has been associated with high carbohydrate meals, strenuous exercise, alcohol intoxication, trauma, infection, stress, and medications. A high glucose load, insulin infusion, and exercise test could induce paralysis and weakness during the post-exercise resting period [6]. Our patient described three episodes of periodic paralysis, each of which can be associated with common precipitating factors. Unlike familial hypokalemic periodic paralysis, TPP can only happen during a hyperthyroid state [7].

Different mechanisms are hypothesized to be involved in causing the hypokalemic state that leads to paralysis. The three original and most commonly cited thoughts are related to Na-K ATPase activity stimulation by thyroid hormone or hyperadrenergic activity and the hyperinsulinemia-induced intracellular influx of potassium. Recent studies have supported the genetic loss of function mutations in inward rectifying potassium channels associated with TPP. This, coupled with increased Na-K ATPase activity, can lead to a cycle of extreme hypokalemia, as seen in periodic paralysis [8].

TPP is a reversible and controllable complication of thyrotoxicosis once diagnosed. Management is divided into phases: acute crisis stabilization and maintenance or definitive treatment. The acute phase is corrected by aggressive potassium repletion and initiation of a non-specific beta-adrenergic blocker such as propranolol. Since there is no change in total body potassium, correction of the hypokalemia needs to be monitored to prevent rebound hyperkalemia. Serial EKGs can also be taken to ensure the changes in potassium, hypokalemia, or hyperkalemia. Potassium supplementation has no proven benefits in preventing future TPP events [9]. Propranolol is recommended in cases refractive to potassium supplementation but can also be used in initial treatment along with lower-dosed potassium to reduce the risk of rebound hyperkalemia [9]. Long-term treatment and prevention are to establish hyperthyroidism control. Propranolol should be continued until a euthyroid state is achieved, along with antithyroid medications such as methimazole or propylthiouracil, radioactive iodine ablation, or thyroidectomy.

## Conclusions

Our patient with no history of hyperthyroidism presented weakness of the upper and lower extremity, which are uncommon presentations of TPP. It supports that TPP should be considered in all young adult Asian males with acute paralysis, and thyroid function tests should be conducted even without thyrotoxic symptoms. Although uncommon in North America, human immigration has been predicted to result in an increased incidence of TPP in the future. Our report emphasizes the importance of TPP education to providers, as this will be more prevalent and relevant to our diversifying patient population.
